# The reduction of abiotic stress in food crops through climate-smart mycorrhiza-enriched biofertilizer

**DOI:** 10.3934/microbiol.2024031

**Published:** 2024-08-21

**Authors:** Mohammad Zahangeer Alam, Malancha Dey (Roy)

**Affiliations:** 1 Department of Environmental Science, Faculty of Agriculture, Bangabandhu Sheikh Mujibur Rahman Agricultural University (BSMRAU), Gazipur-1706, Bangladesh; 2 Progyan Foundation for Research and Innovation (PFRI), Research Organ of the South Asian Forum for Environment (SAFE), India

**Keywords:** arbuscular mycorrhizal fungi, biofertilizers, salinity, drought, heavy metals, crops

## Abstract

Climate change enhances stress in food crops. Recently, abiotic stress such as metalloid toxicity, salinity, and drought have increased in food crops. Mycorrhizal fungi can accumulate several nutrients within their hyphae through a symbiotic relationship and release them to cells in the root of the food crops under stress conditions. We have studied arbuscular mycorrhizal fungi (AMF)-enriched biofertilizers as a climate-smart technology option to increase safe and healthy food production under abiotic stress. AMF such as *Glomus sp*., *Rhizophagus sp*., *Acaulospora morrowiae*, *Paraglomus occultum*, *Funneliformis mosseae*, and *Claroideoglomus etunicatum* enhance growth and yield in food crops grown in soils under abiotic stress. AMF also works as a bioremediation material in food crops grown in soil. More precisely, the arsenic concentrations in grains decrease by 57% with AMF application. In addition, AMF increases mineral contents, and antioxidant activities under drought and salinity stress in food crops. Catalase (CAT) and ascorbate peroxidase (APX) increased by 45% and 70% in AMF-treated plants under drought stress. AMF-enriched biofertilizers are used in crop fields like precision agriculture to reduce the demand for chemical fertilizers. Subsequently, AMF-enriched climate-smart biofertilizers increase nutritional quality by reducing abiotic stress in food crops grown in soils. Consequently, a climate resilience environment might be developed using AMF-enriched biofertilizers for sustainable livelihood.

## 1. Introduction

Microbial biofertilizer is an essential part of climate-smart agriculture (CSA). It improves agricultural productivity, farmer incomes, and resilience to climate change, and reduces greenhouse gas emissions [1]. Microbial strains like arbuscular mycorrhizal fungi (AMF) can be used in biofertilizers that improve soil quality and reduce the demand for chemical fertilizers in food crops grown under abiotic stress. Consequently, AMF-enriched fertilizers can be considered climate-smart biofertilizers for increasing biomass growth in food crops grown under climate-change-induced stress conditions [1]. *Ectomycorrhiza*, *Ectendomycorrhiza*, and *Endomycorrhizal* fungi are available in the environment. Arbuscular mycorrhizal fungi (AMF) are known as Endomycorrhiza under the phylum *Glomeromycota* [2].

AMFs are vastly associated with over 80% of plant species through symbiotic relations [3]–[5]. Hyphae of AMF can easily penetrate smaller pores of root cells [6]. They can exchange carbohydrates and minerals between each other inside the roots. AMF hyphae form a branched structure in the root cortex known as arbuscules. These arbuscules work as the functional site of nutrient exchange for increasing plant growth [7]–[10]. AMF receives lipids from food crops for survival [11],[12]. Several food crops (onions, leeks, garlic, carrots, lettuce, cucumbers, lentils, rice, mung beans, peas, tomatoes, and peppers) form symbiotic associations with AMF [13]. AMF increases the availability of nutrients through their hyphal network [3],[14]. This increased nutrient content [15] improves yield and biomass growth under stress conditions in food crops [16]–[19]. For instance, leaves, roots, and shoots increased significantly under stress conditions in AMF-inoculated plants [20]. Consequently, productivity in food crops has increased remarkably under stress conditions [21].

In addition, mycorrhizae increase root surface area for water and nutrient uptake in crops. Plants with mycorrhizal association will have higher efficiency for nutrient absorption, such as nitrogen, potassium, calcium, magnesium, zinc, and copper; and also increase plant resistance to stress [22]. Mycorrhizal fungi can supply phosphate nutrients through hyphae to plant cells [23]. AMF grows widely in the soil to form a well-developed hyphal network that absorbs inorganic phosphorus (Pi) (via fungal high-affinity PiTs). AMF fungus forms arbuscules with coiled hyphae in the root cortex. This structure is enclosed with a plasma membrane and is potentially important to control nutrient transfers between the symbionts. This character of AMF increases phosphorus (P) uptake and plant biomass growth [24].

The extra-radical mycelium (ERM) of AMF can effectively improve nutrient uptake, thus improving plant growth and development [15]. Both macro- and micro-nutrients are increased significantly for plant growth in nutrient-deficient soils through symbiosis [25]. It is believed that AMF improves nutrients and decreases the uptake of Na and Cl, leading to growth stimulation [3],[26]. In this regard, mycorrhizae can be used as a nutrient stimulator in the farmer's field. Literature showed that crop yield improved by more than 50%, and the farmers' income increased by 61% with the recommended doses of chemical fertilizer and mycorrhizal biofertilizer compared to chemical fertilizer alone [27]. Mycorrhizal association in plant roots resists root and collar rot diseases caused by other fungi. It can be used together with other agricultural chemicals. Mycorrhizae are tolerant to several chemical substances; for example, pesticides such as endrin, chlordane, methyl parathion, and methomyl carbofuran. In this regard, mycorrhizae containing biofertilizers are highly recommended in food crops grown under abiotic stress [22].

Environmental hazards like drought, salinity, metalloid toxicity, and disease epidemics have increased significantly. Climate-smart AMF-enriched biofertilizers can be used as an effective tool in reducing abiotic stress in food crops [20],[21]. Research has shown that climate-smart biofertilizers using *Acaulospora morrowiae*, *Paraglomus occultum*, *Funneliformis mosseae*, *Rhizophagus clarus*, and *Rhizophagus intraradices* increase yield, chlorophyll, carotenoids, catalase (CAT), ascorbate peroxidase (APX), and minerals, and reduces hydrogen peroxide (H_2_O_2_) and malondialdehyde (MDA) in tomatoes (*Solanum lycopersicum* L.) grown in soil under drought stress [28]–[29]. However, drought reduction in plants using AMF is a complex process [30].

AMF is a dispersed fungus [31]–[34]. It expands the availability of water [35], increases the gas changes abilities in the host plant [30], changes root morphology [34], controls hormones [36], and decreases ROS [37]. Thus, reduces the hostile environments for food crops. Glomalin-related soil proteins (GRSP) are produced by AMF which work as a glue and improve water-holding capacity [38]. Also, AMF colonization significantly increases the accumulation of auxins (IAA), gibberellic acid (GA), and jasmonic acid (JA), which improves plant growth under drought stress [39]–[40]. AMF improves anti-oxidant activities, regulates osmolytes, and increases the photosynthetic performance under drought stress [41].

Salinity creates an antagonistic environment for crop production. Twenty-six percent of salinity has increased over the last three decades in the coastal region of Bangladesh [42]. Globally, more than 3 to 6% of soils are altered by salinity. These saline soils are extremely noticeable [43]. AMF can be recommended for decreasing salinity levels in food crops [44],[45]. AMF provides numerous ways to alleviate salinity stress. For instance, AMF can improve nutrient uptake, osmotic balance, antioxidant activities, and hormonal balance in plants grown in saline soils [45]. AMF reduces reactive oxygen species (ROS) in food crops by alleviating salinity [46]. It also increased the activities of peroxidase (POD), superoxide dismutase (SOD), ascorbate peroxidase (APX), and catalase (CAT) in food crops [40],[47]. It is strongly demonstrated that *Rhizophagus irregularis* SA and *Funneliformis mosseae* BEG95 (1:1) can alleviate salinity stress. In contrast, biofertilizers are prepared using alive cells of microbes that increase available nutrients for plants in saline soil [48]. AMF effectively enhances the salinity tolerance of plants by enhancing leaf gas exchanges, peroxidase, catalase, and superoxide dismutase activities, decreasing malondialdehyde contents, increasing the P/N ratio, and absorbing less Na^+^ and more Ca^2+^ in their tissues [48].

In contrast, arsenic (As) is a deadly metalloid [49]. More than 60 million people are at risk of arsenic poisoning in Bangladesh [50]. A hundred million individuals are often in contact with As from potable water. The situation is overwhelming in South Asia [3]. Human and natural activities are responsible for the release of As into the environment. Groundwater, mineral ore, geothermal processes, and pesticides are the main sources of As [51]–[54]. Literature has shown that As could contribute to about 30% of the total As ingestion in food sources [54]. AMF remarkably reduces As in lentil plants grown at 8 and 45 mg kg^−1^ As soils [21]. The extended hyphal network of AMF reduces As toxicity in plants by modifying the metal acquisition [21].

AMF decreases As phytoavailability by stabilizing As through mycelium and glomalin [34],[55]. Mycelium forms a network in the soil, effectively immobilizing and trapping As to prevent its uptake by plants. In addition, glomalin is a glycoprotein produced by AMF, which reduces the availability of arsenic [56]. These mechanisms, adopted by AMF to stabilize As, significantly contribute to lowering its potential impact on plants, enhancing agricultural sustainability, and mitigating the risks associated with As contamination. It can be well-defined that AMF is a significant element for nutrients and bioremediation of As in food crops [57].

AMF is an obligate biotroph that exchanges mutual benefits with plants. In this context, AMF can be considered as a natural biofertilizer. Although, naturally AMF richness can represent an effective substitute for conventional fertilization practices [58]. The production of AMF inoculum is highly laborious due to its obligate biotrophic nature. However, endomycorrhiza-enriched biofertilizers have already been explored in different countries. The mycorrhizae biofertilizer was used in economic crops such as fruit trees. Now, this biofertilizer can be used for food crops grown in stress soils. Therefore, AMF-enriched climate smart biofertilizers might be developed to increase the nutritional quality and antioxidants by reducing abiotic stress in food crops grown in soils.

## 2. AMF effectiveness differs with climatic and soil conditions

Arbuscular mycorrhizal fungi are widely characterized by geographical variability [59]. Soil type is a major factor in shaping AMF communities [60]. AMFs are variable in acidic and calcareous soils [61]. Literature shows that AMF communities can be affected by organic carbon and nitrogen contents in soils [62]. AMF with plants changes by edaphic factors such as nitrogen (N), phosphorus (P), magnesium (Mg), and potassium (K) contents and soil texture [36]. Still, comparatively little evidence is known about the impact of climate variables on AMF communities. The density and diversity of the AMF population were positively correlated with rainfall during the growing season [60]. Soil factors (especially pH, N, Zn, and Cu) mostly affected the variation in AMF communities associated with *Chenopodium ambrosioides*, while geographic and climate factors affected smaller variations [63]. Temperature, precipitation, N, and K strongly affected the abundance of AMF species associated with *Robinia pseudoacacia* [64]. AMF colonization was lower in sand than in gypsum or limestone soils and was largely explained by environmental factors. Soil physical stress also interrupted the variability of AMF with root colonization [64].

## 3. Inorganic fertilizers vs. biofertilizers

Inorganic fertilizers are classified based on the content of the nutrient element and their physical form can vary (solid or liquid) [65]. The most common traditional fertilizers include potassium (K), nitrogen (N), and phosphorus (P). Some fertilizers contain single nutrients that may be known as simple fertilizers. They have active ingredients that are easily soluble in water, rapidly decomposable, and easily absorbable by roots [65].

In contrast, biofertilizers are prepared using alive cells of microbes that increase nutrient solubility or plant access to nutrients. Biofertilizers are one of the vital components in integrated nutrient management in terms of cost-benefits and environmental friendliness. Several microorganisms are used for the production of biofertilizers. Table 1 shows the development of different types of biofertilizers using microorganisms such as algae, fungi, or bacteria [66]. Bacterial biofertilizers are used in crops as nitrogen fixers, symbiotic and non-symbiotic associative, and phosphate solubilizers (Table 1). Fungal biofertilizers might have different characteristics such as phosphate solubilizers, non-symbiotic, nutrient mobilizers, and symbiotic. Algal biofertilizers may have symbiotic, non-symbiotic, and nitrogen-fixing characteristics in food crops (Table 1).

**Table 1.** Biofertilizers and their character with examples are described below [66].

Types	Character	Example
*Bacterial biofertilizer*	Nitrogen Fixer	This type of biofertilizer contains bacteria that can fix nitrogen. The bacteria produce nodules in the roots of the leguminous crops and add nitrogen to the soil. Free-living bacteria also fix the nitrogen from the atmosphere.
	Symbiotic	*Mesorhizobium*, *Azorhizobium*, *Sinorhizobium*, *Allorhizobium*, *Bradyrhizobium*, *Rhizobium*, etc.
	Associative	*Herbaspirillum*, *Azospirillum*, etc.
	Non-symbiotic	*Azotobacter*, *Derxia*, *Rhodospirillum*, *Rhodopseudomonas*, *Chromatium*, *Beijerinckia*, *Acetobacter*, etc.
	Phosphate Solubilizer	This type of biofertilizer fixes phosphorous through phosphorus-solubilizing microorganisms. Phosphorus is converted into a soluble form by organic acids and enzymes.
	Non-symbiotic	*Pseudomonas striata*, *Bacillus pseudomonas*, *Bacillus circulans*, etc.
*Fungal Biofertilizer*	Phosphate Solubilizer	This biofertilizer comprises fungi. The mechanism is also the same as phosphate solubilizer biofertilizer.
	Non-symbiotic	*Penicillium*, *Aspergillus*, *Trichoderma*, etc.
	Nutrient Mobilizer	This biofertilizer transfers nutrients such as phosphorus from the soil to the cortical cells of the roots. They also perform as carriers of nutrients.
	Symbiotic	Arbuscular mycorrhizal fungi (AMF)
*Algal Biofertilizer*	Nitrogen Fixer	This type of biofertilizer contains algae that can fix nitrogen.
	Symbiotic	Blue-green algae or *cyanobacteria*
	Non-symbiotic	Azolla

However, arbuscular mycorrhizal fungi (AMF) can be applied as biofertilizers [58]. The tree-shaped structures, arbuscules, and fungal hyphae are used in AMF-enriched biofertilizers [3],[5],[6]. In addition, the extended extraradical mycelia and hyphae of AMF increase phosphorus, nitrogen, copper, and zinc under stress conditions [6]. Consequently, AMF might reduce the demand for chemical fertilizer in crop fields [58]. AMF is undoubtedly promising in sustainable farming for its various beneficial purposes such as augmented productivity, nutrient uptake, plant biomass, and yield. Consequently, AMF increases healthy foods for human beings [58]. Table 2 shows that AMF species of *Glomus mosseae*, *Rhizophagus irregularis*, *Glomus intraradices*, *Acaulospora morrowiae*, *Paraglomus occultum*, *Funneliformis mosseae*, *Rhizophagus clarus*, and *Rhizophagus intraradices* enhance biomass growth, yield, antioxidant activities, and mineral and bioactive compounds under abiotic stress in food crops [28],[67]–[69]. *Glomus mosseae* reduces arsenic stress in lentils, mung bean, and pea crops [70]. AMF species of *Glomus etunicatum*, *Glomus intraradices*, *Glomus mosseae*, and *Claroideoglomus etunicatum* reduce salinity stress in rice and cucumber crops [40],[71].

**Table 2.** AMF reduces abiotic stress in food crops grown in soils.

Abiotic Stress	Host	AMF	Benefits	References
Drought	*Glycine max* L.	AMF mixed	It boosted proline, photosynthesis, leaf area, growth, and biomass production	[67]
Drought	*Triticum aestivum*	*Glomus mosseae*	Reduced osmotic damage, increased chlorophyll, antioxidants, ascorbic acid, and nutrients	[68]
Drought	*Lactuca sativa, Solanum lycopersicum*	*Rhizophagus irregularis*, *Glomus intraradices*	Increased biomass and abscisic acid (ABA) accumulation	[69]
Metal-general	*Sesbania rostrata*	*Glomus mosseae*	Enhanced the formation of nodules with root, and increased N and P uptake	[72]
Arsenic	*Lens culinaris*, *Vigna radiata*, *Pisum sativum*	*Glomus mosseae*	Increased biomass and antioxidant	[19]–[21],[49],[70],[73]
Drought	*Solanum lycopersicum*	*Acaulospora morrowiae*, *Paraglomus occultum*, *Funneliformis mosseae*, *Rhizophagus clarus*, *and Rhizophagus intraradices*	Increased biomass and antioxidant activities	[69]
Salinity	*Cucumis sativus*	*Glomus etunicatum*, *Glomus intraradices*, *Glomus mosseae*	Increased biomass, photosynthetic pigment, and antioxidant enzymes	[40]
Salinity	*Oryza sativa L*.	*Claroideoglomus etunicatum*	Improved yield, photosynthetic rate, and stomatal conductance	[71]

### 3.1. Comparative analysis between inorganic and mycorrhizae-enriched biofertilizer

The plant height, grain weight, and yield were 125.73 cm, 151.62 g, and 3536.83 kg ha^−1^ with the treatment of mycorrhiza and recommended doses of chemical fertilizers. In contrast, the application of mycorrhiza and rhizobium showed a thousand-grain weight, and yield was 167.19 g and 4321.41 kg ha^−1^, respectively, in cowpea crops. Mycorrhizae-enriched biofertilizers increase yield by 23% compared to chemical fertilizers [74]. AMF and nitrogen-fixing bacteria have been widely used to improve soil fertility [75]. Also, this type of biofertilizer plays a crucial role in plant metabolism and nutrient availability, facilitating nutrient uptake from the soil [76]. The symbiotic association of rhizobium species with legumes promotes biological nitrogen fixation, phosphate solubilization, and Indole-3-acetic acid (IAA). AMF-containing biofertilizers enhance nutrient mineralization and the root area of crops [77]. In addition, AMF-enriched biofertilizer interacts with other microorganisms in the rhizosphere. It enhances Zn, Cu, Fe, Mn, and other nutrient uptake by expanding the network of hyphae in their cells. AMF improves the storage of carbon and nutrients and provides a favorable habitat for the survival and development of soil microorganisms. AMF also reduces soil-borne diseases, including *Aphanomyces*, *Cylindrocladium*, *Fusarium*, *Macrophomina*, *Phytophthora*, *Pythium*, *Rhizoctonia*, *Sclerotinium*, *Verticillium*, and *Thielaviopsis sp*. [78]. *G*. *intraradices* and *G*. *mosseae* improved K absorption in maize crops. This K-solubilizing AMF improves the growth of cotton, rape, pepper, cucumber, sorghum, wheat, tomato, chili, sudan grass, and tobacco [79]. Therefore, AMF-enriched biofertilizers are economically viable and environment friendly. Soil health and crop productivity also improved using AMF. It could be applied as a supplementary substance with chemical fertilizers [80]. AMF also reduces the demand for phosphorus fertilizers [81]. Incessant application of chemical fertilizers and pesticides creates environmental problems for soil, plants, and human health [82]. AMF enhances nutrients that augment photosynthate production [83],[84]. For example, biomass growth and mineral contents were higher in AMF-inoculated plants [85].

**Figure 1.**
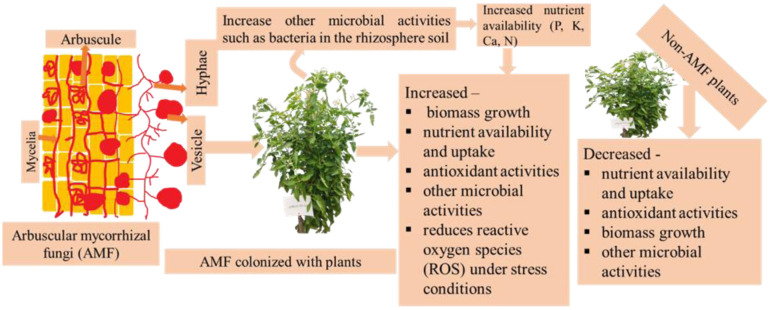
AMF increases other microbial activities and nutrient availability in the rhizosphere soil (adapted from [88]).

Arbuscules of AMF are highly helpful for increasing nutrients, carbon, and phosphorus-containing compounds, finally improving the growth of host plants [86]. It is also detected that AMF maintains P and N uptake in plants for their development under stress conditions. AMF can reduce the demand for chemical fertilizers by up to 50% during crop production. Figure 1 shows that AMF increases biomass growth and microbial activities in soils compared to non-AMF plants [87],[88].

## 4. Development of AMF-enriched biofertilizers

AMF-enriched biofertilizers can be prepared using rhizosphere soils, and root-containing spores, hyphae, mycelium, vesicles, and arbuscules [58]. AMF production is easier than other methods through greenhouse experiments. The purity of the AMF strain is a major issue in developing mycorrhizae-enriched biofertilizers. *In vitro* is the more recognized method for pure culture of AMF [89]. Quite a lot of companies around the world are producing AMF spores. Accordingly, the production of mycorrhizal inoculants has been increasing globally for the last decades. Generally, the suppliers may have their AMF products mostly within their territories. Currently, ectomycorrhizal fungi are exported by a couple of multinational companies globally. This type of mycorrhizal inoculum is used in trees, shrubs, and precious fruit trees.

**Figure 2.**
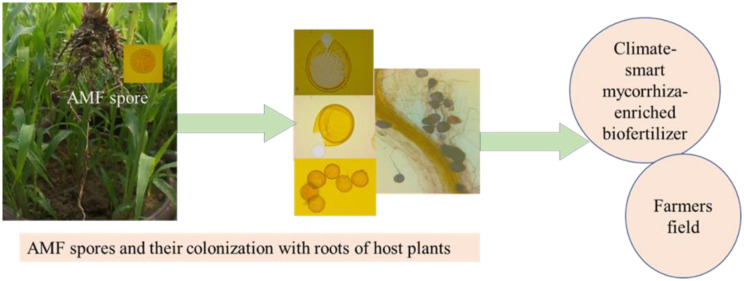
Climate-smart technology: Arbuscular mycorrhizal fungi (AMF)-enriched biofertilizer.

Also, AMFs are applied in vegetables, forests, and ornamental trees under abiotic stress [90]. AMF inoculation is highly used in food crops grown under drought stress. However, the production of AMF inocula with climate-smart technology is rarely visible; but, it is recognized that mycorrhizal fungi flourish crop yield under stress soils. However, quantity and genetic diversity may impact the colonization of AMF with host plants [91]. It is challenging to judge the cost-effectiveness of the AMF product and its rate [58]. However, inspection of the AMF inoculant is not easy due to its fundamentally multifaceted heritable structure [58]. So, molecular techniques are needed to characterize them [58]. Abiotic stress is focused on the crops' responses in the field crops [58]. Meta-analyses have been suggested for growth responses to AMF inoculations, based on the greenhouse and field conditions [91]. AMF has already been used in manufacturing biofertilizers for their constructive response in terms of stress. The collection of AMF spores, culturing them with host plants, identification, and use against stress in food crops are the main procedures for the preparation of AMF-enriched climate smart biofertilizer. The methods for the preparation of AMF-enriched biofertilizer are shown in Figure 2.

## 5. AMF-enriched biofertilizers reduce abiotic stress

**Figure 3.**
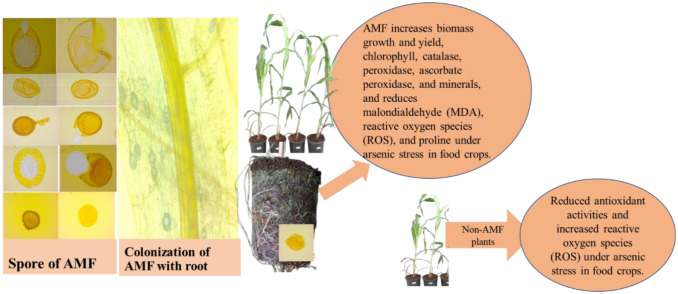
Arbuscular mycorrhizal fungi (AMF) improve photosynthetic pigments and antioxidant activity under arsenic stress in food crops (adapted from [19]).

Arsenic (As) stress has been a global problem in food crops for the last three decades [92]. Arsenic in soils is one of the most important abiotic stresses that reduce plant biomass growth and the quality of food production. Arsenic reduces growth, pigment, total chlorophyll, catalase (CAT), and ascorbic acid content in food crops [93]. For this reason, AMF is recommended to increase chlorophyll and CAT activity, and reduce oxidative stress in As-stressed crops (Figure 3). AMF enhances antioxidant defense mechanisms and the nutritional quality of food crops grown in As soils [20]. AMF has great potential in reducing As transfer in biomass and grains of food crops [21]. More precisely, the As in grains of food crops decreased by 57% with AMF application [70]. Arsenic (As) is stored in the vacuole through fungal hyphae [94],[95]. This hypha of AMF improves the growth, yield, and nutrient status of food crops under As stress [96]–[99].

**Figure 4.**
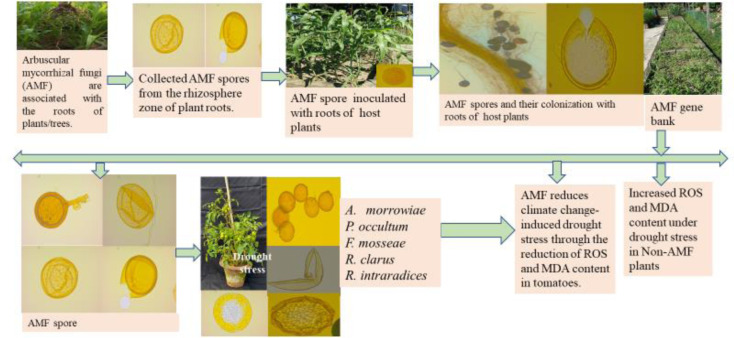
Arbuscular mycorrhizal fungi increase biomass growth and antioxidants and reduce climate change-induced drought in plants.

Under drought stress, AMF also increases nutritional quality and antioxidant activities in food crops grown in soil (Figure 4). Many studies show that AMF reduces drought stress in food crops [100]. The plant's tolerance to drought increased using the extra-radical hyphae of the AMF [101]. As a result, biomass production increased under drought stress [102],[103]. Gas exchange, leaf water potential, stomatal conductance, and transpiration rate are increased through the symbiosis of AMF [104]–[106]. Photosynthesis in C3 (*Leymus chinensis*) and C4 (*Hemarthria altissima*) plants increased using AMF under stress conditions. The biomass growth with AMF-treated plants was significantly higher than that of the control in tomatoes (non-AMF). In AMF-treated tomatoes, CAT and APX increased by 42% and 66%, respectively, compared to non-AMF under drought conditions. MDA and H_2_O_2_ (ROS) in AMF-treated tomato plants were also reduced by 50% and 2% compared to the control. Minerals of tomato fruits improved by 36% with AMF treatment than that of the control [28]. AMF significantly enhanced drought tolerance and biomass production in plants over the activity of N metabolizing enzymes [107].

**Figure 5.**
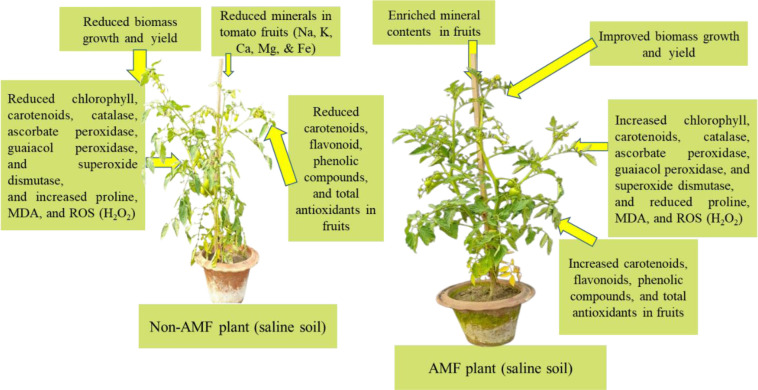
Differential response of an AMF and non-AMF plant under salinity stress (adapted from [119],[121]).

Global food security is affected due to soil salinity [108]. Reactive oxygen species (ROS) are enhanced in food crops grown in saline soils [109],[110]. The biomass growth, photosynthetic rate, stomatal conductance, leaf water potential, and water use efficiency enhanced using AMF in plants grown under salinity stress [111],[112]. AMF also enhanced gas exchange, leaf area index, fresh and dry biomass, and chlorophyll content in food crops under saline conditions [113]–[115]. In addition, P, N, Ca, and K were higher in the AMF-treated plants under salt-stress conditions [116]–[118]. Malondialdehyde (MDA), superoxide dismutase (SOD), proline, peroxidase (POD), superoxide dismutase (SOD), and catalase (CAT) are changed in plants through AMF under salinity stress. Therefore, the effect of AMF on plant growth and physiology is more notable under salinity stress [119]. The production of AMF inoculum is a bit tough due to its obligate symbiotic behavior with host plants. As a consequence, a methodology is needed for the production of AMF on a large scale. However, AMF-enriched climate-smart biofertilizers might be developed to improve the nutritional quality and antioxidants in food crops grown in soils under abiotic stress conditions (Figure 5).

It is already clear that AMF increases biomass growth, yield, and antioxidant activities under arsenic, drought, and salinity stress in food crops grown in soil [28]. Therefore, AMF-enriched biofertilizers might be recognized as climate-smart biofertilizers.

## 6. Future outlook and research gaps as an emerging technology

Global food production is required to double by 2050 to feed the increasing human population. Synthetic fertilizers in agriculture have lost soil quality due to many environmental consequences. Biofertilizers are recognized as an advanced approach to reducing environmental stress and maintaining sustainability in agriculture. There is much evidence that beneficial microbes improve soil productivity and quality. Of many microbial contestants, AMF has been shown potential to be used as a biofertilizer due to its numerous benefits. Plant growth and yield, nutrient availability, water-holding capacity, and disease resistance increased by the application of AMF-enriched biofertilizers. In addition, AMF could also play a role in controlling soil erosion, improving the initial growth of seedlings, remediating soil pollutants, and eradicating harmful organisms. In the future, a diverse pool of AMF species should be used in crop fields based on their host and environmental preferences. So, the selection of the best inoculum is highly recommended for crops. Technologies and protocols should be used to select the effective inocula. Maintaining quality control of products is also significantly important to commercializing AMF inoculants to meet the needs of the farmers. Once these challenges are addressed properly, AMF has more potential as a natural biofertilizer in future agriculture. This AMF-enriched biofertilizer will be used in crop fields like precision agriculture, which reduces the demand for chemical fertilizers and the impacts of climate change in crop fields. Finally, a climate resilience environment will be developed by AMF-enriched biofertilizers in the crop field [120],[121].

Climate change induces an unexpected environment for crop cultivation. The symbiosis of microbial inoculants depends on the crop species, native microbial communities, soil type, and nutrient availability in soils. A study is needed on how the pathogen, temperature, rainfall, and antimicrobial activities affect the efficacy of AMF inoculant in mycorrhiza-enriched biofertilizers. Further field trials are essential to understand the factors hindering consistently positive plant responses to AMF inoculants and help farmers determine whether they are appropriate for their system. The viability of hyphae, mycelia, and spores in the mycorrhiza-enriched biofertilizers depends on the temperature. Further study is needed about the viability of AMF spores under specific temperatures.

## 7. Conclusions

A few researches have been done regarding the positive effect of AMF in increasing plant biomass under abiotic stress. Still, the role of AMF on plant growth is unknown in stressful environments. AMF has been mainly used as a valuable material for increasing nutrients in food crops. Recently, AMF can effectively reduce salinity, drought, and arsenic stress in food crops, thus increasing the yield of crops and vegetables. Therefore, AMF practice is tremendously important for its consistent sustainability in modern agricultural systems. AMFs must be explored at all levels to prepare as a natural climate-smart biofertilizer to reduce abiotic stress in sustainable agriculture. AMF-enriched biofertilizer may be applied to field crops. Thus, this type of fertilizer will be able to make a mutual relationship with food crops to supply them with nutrients under stressful conditions. A quality and regulation framework should be forwarded by an expanded list of authors to ensure that microbial products contain viable propagules, and an absence of pathogens, and are packaged with labels describing their contents and nutrient adjuncts. The benefits of introducing AMF into agricultural soils will be more predictable, which will turn them into more reliable and less environmentally damaging methods of improving crop productivity.

## Use of AI tools declaration

The authors declare they have not used Artificial Intelligence (AI) tools in the creation of this article.
